# A machine-learning based model for automated recommendation of individualized treatment of rifampicin-resistant tuberculosis

**DOI:** 10.1371/journal.pone.0306101

**Published:** 2024-09-06

**Authors:** Lennert Verboven, Steven Callens, John Black, Gary Maartens, Kelly E. Dooley, Samantha Potgieter, Ruben Cartuyvels, SMARTT team, Kris Laukens, Robin M. Warren, Annelies Van Rie

**Affiliations:** 1 Torch Consortium FAMPOP Faculty of Medicine and Health Sciences, University of Antwerp, Antwerpen, Belgium; 2 Department of Computer Science, ADReM Data Lab, University of Antwerp, Antwerpen, Belgium; 3 Department of Internal Medicine & Infectious diseases, Ghent University Hospital, Ghent, Belgium; 4 Department of Internal Medicine, University of Cape Town and Livingstone Hospital, Port Elizabeth, South Africa; 5 Department of Medicine, Division of Clinical Pharmacology, University of Cape Town, Cape Town, South Africa; 6 Department of Medicine, Vanderbilt University Medical Center, Nashville, TN, United States of America; 7 Department of Internal Medicine, Division of Infectious Diseases, Faculty of Health Sciences, University of the Free State, Bloemfontein, South Africa; 8 Department of Computer Science, KU Leuven, Belgium; 9 DSI-NRF Centre of Excellence for Biomedical Tuberculosis Research, SAMRC Centre for Tuberculosis Research, Division of Molecular Biology and Human Genetics, Stellenbosch University, Cape Town, South Africa; 10 The Aurum Institute, Johannesburg, South Africa; 11 Department of Medical Microbiology, Universitas Academic Laboratory, National Health Laboratory Service, University of the Free State, Bloemfontein, South Africa; 12 Department of Medical Microbiology, University of the Free State, Bloemfontein, South Africa; 13 Free State Department of Health, TB, DR-TB & CDC Directorate, Bloemfontein, South Africa; Boston University School of Public Health, UNITED STATES OF AMERICA

## Abstract

**Background:**

Rifampicin resistant tuberculosis remains a global health problem with almost half a million new cases annually. In high-income countries patients empirically start a standardized treatment regimen, followed by an individualized regimen guided by drug susceptibility test (DST) results. In most settings, DST information is not available or is limited to isoniazid and fluoroquinolones. Whole genome sequencing could more accurately guide individualized treatment as the full drug resistance profile is obtained with a single test. Whole genome sequencing has not reached its full potential for patient care, in part due to the complexity of translating a resistance profile into the most effective individualized regimen.

**Methods:**

We developed a treatment recommender clinical decision support system (CDSS) and an accompanying web application for user-friendly recommendation of the optimal individualized treatment regimen to a clinician.

**Results:**

Following expert stakeholder meetings and literature review, nine drug features and 14 treatment regimen features were identified and quantified. Using machine learning, a model was developed to predict the optimal treatment regimen based on a training set of 3895 treatment regimen-expert feedback pairs. The acceptability of the treatment recommender CDSS was assessed as part of a clinical trial and in a routine care setting. Within the clinical trial setting, all patients received the CDSS recommended treatment. In 8 of 20 cases, the initial recommendation was recomputed because of stock out, clinical contra-indication or toxicity. In routine care setting, physicians rejected the treatment recommendation in 7 out of 15 cases because it deviated from the national TB treatment guidelines. A survey indicated that the treatment recommender CDSS is easy to use and useful in clinical practice but requires digital infrastructure support and training.

**Conclusions:**

Our findings suggest that global implementation of the novel treatment recommender CDSS holds the potential to improve treatment outcomes of patients with RR-TB, especially those with ‘difficult-to-treat’ forms of RR-TB.

## Introduction

Tuberculosis (TB) continues to be a global public health problem with about 10 million new cases and 1.4 million TB deaths annually [1]. The ‘End TB Strategy’ of the World Health Organization (WHO) aims to reduce new TB cases by 90% and TB deaths by 95% by 2035. A major challenge to achieve these goals is the occurrence of about 450,000 new cases of rifampicin resistant TB (RR-TB) annually [1], cases which are difficult, complex, and costly to treat.

All patients diagnosed with RR-TB should start a standard RR-TB treatment regimen. Since 2019, a 9-12 month all-oral 7-drug RR-TB regimen is recommended for patients who have not been previously treated for ≥ 1 month with bedaquiline, clofazimine or linezolid and in whom resistance to fluoroquinolones is unlikely or excluded [2]. Otherwise, a longer (18 to 20 months) regimen containing all three group A agents (levofloxacin/moxifloxacin, bedaquiline, linezolid) and at least one group B agent (clofazimine, cycloserine/terizidone) should be administered. Group C agents (ethambutol, delamanid, pyrazinamide, imipenem-cilastatin/meropenem, amikacin/streptomycin, ethionamide, prothionamide, *para*-aminosalicylic acid) can be added to ensure the regimen contains at least four drugs to which the *Mycobacterium tuberculosis* (*Mtb*) strain is most likely susceptible [2]. In 2022, a 6-month regimen containing bedaquiline, pretomanid, linezolid, and moxifloxacin (BPaLM) was endorsed by the World Health Organization (WHO) for treatment of RR-TB and a 6-9 month regimen of bedaquiline, pretomanid, and linezolid (BPaL) for fluoroquinolone resistant RR-TB [3], but pretomanid was not yet registered or available in South Africa when the study was performed.

Ideally, the standard RR-TB treatment regimen should be individualized when drug susceptibility test (DST) results identify the presence of resistance to one or more drugs included in the regimen. Because DST assays can take months, this is not always implemented. For example, between November 2012 and December 2013 treatment was individualized in only 57% of pre-XDR and 68% of XDR-TB patients [4]. Molecular methods are increasingly used for rapid DST [5]. Line probe assays (LPA) are used in many countries for detection of resistance to isoniazid, injectable drugs and fluoroquinolones in people diagnosed with RR-TB. More recently, the Xpert MTB/XDR assay for the diagnosis of resistance to isoniazid, fluoroquinolones, ethionamide, and injectable drugs and a LPA for detection of resistance to pyrazinamide have been endorsed by the WHO but these are not yet implemented in most countries [6]. Rapid tests for ethambutol, terizidone, cycloserine, bedaquiline, clofazimine, linezolid, delamanid, pretomanid and PAS do not exist. In the absence of a complete drug resistance profile, it is challenging to correctly compose an individualized RR-TB treatment regimen.

Next generation sequencing (NGS), using a targeted or whole genome sequencing (WGS) approach, can provide a comprehensive genomic drug resistance profile. In 2018, the WHO endorsed NGS for surveillance but not for clinical care [7]. Some public health laboratories in high income, low RR-TB burden settings have integrated WGS into the routine patient management [8–10]. In low- and middle-income countries, the use of NGS remains limited to research institutions and reference laboratories [11] because of limited bioinformatics expertise, scarce sequencing infrastructure, expertise required for the interpretation of sequencing data, and challenges in translating a drug resistance profile into the optimal individualized treatment regimen [12, 13]. Increased automation could facilitate the use of NGS data for individualized RR-TB treatment and increase the patient benefit that could arise from the scientific advances made.

In this paper, we describe the development of a computational model using machine learning methods to create a clinical decision support system (CDSS) that automates the translation of NGS data into an individualized RR-TB treatment regimen recommendation with its accompanying user-friendly web interface and present the results of the acceptance assessment of the tool by physicians in South Africa.

## Methods

### Ethics approval and consent to participate

The research was performed in accordance with the Declaration of Helsinki. Collection of the clinical strains and the determination of the genotypic drug resistance profile was obtained from the Human Research Ethics Committee of the University of the Witwatersrand in South Africa and the Institutional Review Board of the University of North Carolina at Chapel Hill in the United States for the training dataset, and from the Stellenbosch University Health Research Ethics Committee for the validation dataset. Participants in the training dataset gave verbal informed consent by phone (recorded) as approved by the Human Research Ethics Committee of the University of the Witwatersrand in South Africa and the Institutional Review Board of the University of North Carolina at Chapel Hill in the United States. The Research Ethics Committee or Institutional Review Board of Human Research Ethics Committee of the University of the Witwatersrand in South Africa and the Institutional Review Board of the University of North Carolina at Chapel Hill in the United States waived the need of informed consent for patients who had died or were lost to follow-up from TB care prior to study enrolment and could not be contacted despite multiple attempts. For the validation dataset, the Stellenbosch University Health Research Ethics Committee waived the need for informed consent. The study titled "A personalized recommendation system for Whole Genome Sequencing-based individualized treatment for drug resistant tuberculosis" reported in this manuscript was approved by the Ethics Committee of the University Hospital Antwerp and the University of Antwerp.

### Development of the RR-TB treatment recommender CDSS

The development process of the RR-TB treatment recommender CDSS consisted of the assembly of the knowledge base, development of a heuristic model prototype, feedback harvesting from experts, application of machine learning methods to analyze the feedback, assessment of the CDSS performance, and external validation [14]. The stakeholder group included experts in pathogen genomics (genotype-phenotype associations), pharmacology (drug properties, mechanisms of action, drug-drug interactions, synergy and antagonism between drugs), medicine (treatment of RR-TB in high and low burden settings), and computer and data science. In addition, public health practitioners and patients were included to hear their viewpoints. Stakeholder discussions were held in 2019 and focused on the minimum number of effective drugs to be included in a regimen, the role of an intensive treatment phase, drug toxicity, burden of treatment monitoring, drug properties that define effectiveness, and level of drug resistance.

The key features of individual drugs and treatment regimens were quantified by five experts in 2019, through expert panel discussions guided by review of published and unpublished data. The key features identified by the stakeholders were toxicity, early bactericidal activity (EBA), bactericidal activity, sterilizing activity, mode of administration (oral vs injection), mechanism of drug action, propensity to acquire resistance, and cost (S1 Table). After the first round of feedback, QTc prolongation was added as a separate feature, resulting in nine features to characterize individual drugs in the model. Toxicity was classified for each drug as life threatening, permanent (e.g., hearing loss), short-term with possible effect on adherence (e.g., nausea) or minimal without patient impact (e.g., liver function test abnormality grade 2). Based on a combination of frequency of occurrence and severity, the level of toxicity for each drug was graded on a scale of 1 to 3. QTc prolongation was classified into four categories (none, low, moderate, or high). Early bactericidal activity (EBA), bactericidal activity and sterilizing activity were classified as one of five categories (very low, low, moderate, high, very high). EBA classification was based on the drop in log_10_ colony forming units (CFU) in the first days of treatment observed in experimental EBA studies and bactericidal activity on the drop in log_10_CFU in the first six months of treatment. Sterilizing activity, or the ability to achieve stable cure was defined based on a drug’s ability to prevent relapse in human or animal model studies. The propensity to acquire resistance was classified into four categories (low, moderate, high, and none) based on expert opinion. Mode of administration was defined as either oral or injection (intravenous or intramuscular). All cost for the drugs at recommended dosing was quantified as the cost for one month of treatment in South Africa [15–18].

In the heuristic model prototype all features received equal weight, possible interaction between features were not considered, and all regimen features were normalized to obtain a regimen score with a higher score representing a better regimen.

A training dataset of 129 unique WGS-derived drug resistance profiles was obtained using WGS data and interpretation of variants based on the 2021 WHO catalogue of mutations in Mtb [19]. Of these 129 unique resistance profiles, 119 (92%) were resistant to rifampicin, 106 (82%) to isoniazid, and 29 (22%) to fluoroquinolones from 303 anonymized South African RR-TB (accessed on 2017.10.10) patients was used to solicit feedback from experts. For each unique drug resistance profile, all available four-drug regimens (defined as a regimen not containing any drug to which the *Mtb* isolate is resistant on WGS) were generated by the treatment recommender model, using all drugs that can be prescribed for RR-TB treatment. A sampling function was used to increase the likelihood for feedback on highly ranked (i.e., better) compared to lower ranked regimens. Regimens were replaced to ensure that multiple experts could provide feedback on the same regimen. Experts were asked to state if they would prescribe the proposed regimen to a patient with the specified resistance profile. If they rejected the regimen, the reason for rejection and suggestion of an alternative regimen was requested. After the first round of feedback, experts discussed which features were missing, obsolete, or sub-optimally quantified and revisions to the model were made. After each round of expert feedback harvesting, a random forest classifier using 100 trees with a maximum depth of 10 and default parameters (from the scikit-learn library [20]) was trained and evaluated using the feedback from the experts. After each round of machine learning, a patient level ‘leave-one-out’ cross validation strategy was used to assess model performance [14]. A leave-one-out cross validation at the patient level means that the model was trained on the data for all patients except one. The dataset and was then used to make recommendations for the excluded patient. This analysis was then repeated until all patients were excluded once. This analytic approach was chosen due to the limited amount of data and because observations were not independent of each other. The random forest classifier was then used to predict for each possible treatment regimen the probability that a regimen is classified as ‘good’. Finally, the regimens were ranked according to these probabilities to identify the optimal (highest ranked) regimen. The result of the machine learning analysis was then used to develop the next version of the treatment recommender model. The iterative feedback harvesting process was continued until no further significant improvements were made in the model prediction of a ‘good’ treatment for a specified resistance profile. After 3 rounds of feedback and machine learning, the treatment recommended by the model was accepted by the experts in 89%, 95%, and 95% of the cases, demonstrating no further improvement after the third round. [14]. The machine learning model and its parameters were decided upon before the start of the study and kept fixed throughout it, since the training data had to be iteratively generated through the expert feedback harvesting and we did not want to update the methodology mid study.

The model was then validated using a dataset of 64 unique anonymized WGS-derived drug resistance profiles that were not present in the training dataset (accessed on 2020.01.13) [14].

For the final treatment recommender CDSS, the model was retrained on a dataset of 3895 treatment regimen-expert feedback pairs obtained from 268 unique WGS-derived resistance profiles consisting of 129 unique resistance profiles used during training, the 64 unique resistance profiles used during validation, and 75 unique resistance profiles generated from the 129 profiles excluding pretomanid, high-dose rifampicin, and cycloserine as possible drugs. As the model was to be used in a clinical trial, the model was retrained using all available resistance profile-treatment regimen data with expert feedback to make sure the final model included as much training data as possible. Pretomanid, high-dose rifampicin and cycloserine were excluded at this stage as these drugs were not recommended or used for clinical care in South Africa at the time of model development, due to the cost involved (timewise for the experts providing their feedback) as reevaluating all the original resistance profile-treatment regimen pairs would not be worth the time investment. Additionally, this also allows shows that the exclusion of certain drugs is possible should the clinical setting require it. The treatment recommender model with training data and instructions on usage are available from https://github.com/LennertVerboven/treatment_recommender.

### Development of interactive online web interface

We developed a user-friendly, interactive web application optimized for mobile phone (available from https://github.com/LennertVerboven/treatment_recommender_webapp). The front end was written in JavaScript using the React library. The back end written in Python3, using the Django web development framework, exposes a representational state transfer (REST) application programming interface (API) to which the clients can make secured hypertext transfer protocol secure (HTTPS) requests. The backend implements the connection between the REDCap [21] server containing the patient data and the treatment recommender machine learning model to make individualized treatment recommendations.

The online web interface allows health care workers to log in securely using username and password authentication and then shows a list of their patients. For each patient, the app lists the patient’s demographic information (name, date of birth, clinic, phone number), current treatment regimen, contra-indications based on clinical information, toxicity, stockouts, the WGS-based resistance profile, and the recommended regimen. The physician can then start an interactive process to enter any contra-indications, toxicity, or drug stockouts for any of the drugs included in the recommended regimen, after which an updated treatment regimen recommendation is proposed in real-time. This iterative process is completed once the physician accepts the treatment recommendation. At that time, a pdf report is generated for inclusion in the patients’ paper medical file.

To ensure data security, all data transfers between the front end and the server hosting the webapp used an encrypted connection. All patient data was stored on a secure server at the University of Stellenbosch [21].

### Acceptance assessment of the RR-TB treatment recommender CDSS

The treatment recommender CDSS and its accompanying webapp were implemented as part of the SMARTT pragmatic trial (*Clinicaltrials*.*gov Identifier NCT05017324)* [22]. The trial aimed to determine the effectiveness of a WGS-based drug susceptibility testing strategy to guide individualized treatment for patients diagnosed with RR-TB.

**Fig 1:**
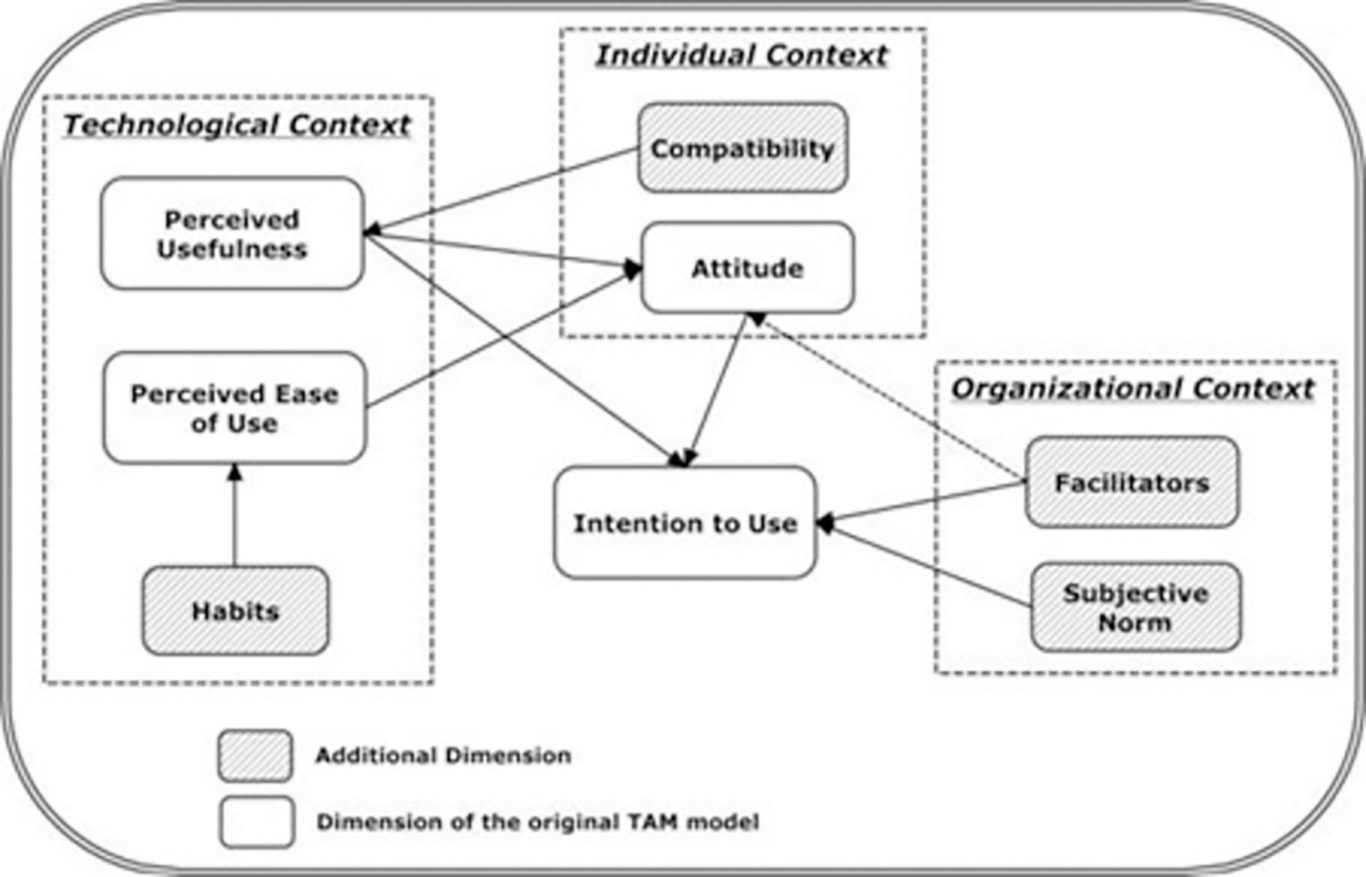
Modified technology acceptance model as created by Gagnon *et al*. [23].

Acceptance of the treatment recommender and its accompanying online app by clinicians caring for trial participants was assessed in three different ways. First, we investigated the proportion of trial participants for whom the treatment proposed by the treatment recommender CDSS was prescribed. Because this is an ongoing trial, we performed this analysis on the first 20 patients randomized to the intervention (WGS) arm. Second, we determined the proportion of patients for whom clinicians would prescribe the regimens proposed by the treatment recommender in their routine practice, i.e., outside of the trial setting. This was done by presenting the treatment regimens recommended by the CDSS for 15 unique resistance profiles to five physicians who had experience with the CDSS in the trial setting and agreed to participate in this assessment. Resistance profiles were selected from those that have been documented in the region where these physicians practice. Physicians were asked if they would prescribe the proposed regimen for a patient receiving routine RR-TB care. Third, we performed a survey to assess the factors that may influence health care workers’ use of the treatment recommender CDSS. The questionnaire (S2 Table) was developed based on the modified technology acceptance model (Fig 1) and consisted of 36 questions investigating eight domains within the technological, individual, and organizational context (Table 1) [23]. Participants were asked to rate each question on a 5-point Likert scale. Responses were transformed into a score ranging from 0 (Strongly disagree) to 4 (strongly agree), except for questions 8 and 32 for which the rating was inverted as these questions were posed negatively. For each domain, the median and range of the sum of the scores were calculated.

**Table 1:** The eight domains of the technology acceptance model and their definitions [23].

Dimension	Definition
Perceived usefulness	Belief that the treatment recommender CDSS will help them care better for patients with RR-TB
Perceived ease of use	The degree to which the physician believes that using the RR-TB treatment recommender CDSS would be effortless
Attitude	Perception of the positive or negative consequences related to adopting the treatment recommender CDSS
Compatibility	The degree of correspondence between the treatment recommender CDSS and existing values, past experiences and needs of potential adopters
Subjective norm	The extent to which an individual believes that people who are important to him or her will approve the physician’s adoption of the treatment recommender CDSS
Facilitators	The degree to which an individual believes that the organizational and technical infrastructure required to support the use of the treatment recommender CDSS exists
Habit	The degree to which the use of the treatment recommender affects habits and behaviors that have become automated
Intention	Willingness of the physician to use the treatment recommender CDSS if it becomes available

## Results

### Selection and quantification of individual drug features

The fluoroquinolones, moxifloxacin and levofloxacin are oral drugs that inhibit DNA gyrase of *Mtb* [24]. Fluoroquinolone toxicity was classified as low (score 1 for levofloxacin, 1.25 for moxifloxacin) given the low (1.2%) incidence of serious adverse effects (SAE) in RR-TB patients receiving fluoroquinolones [25]. QTc prolongation on moxifloxacin is moderate [26] and experts classified the risk of QTc prolongation for levofloxacin as low. The monthly cost is $3.87 for levofloxacin and $8.85 for moxifloxacin [18]. The EBA of fluoroquinolones is very high based on a fall in log_10_CFU of 0.45 for moxifloxacin and 0.53 for levofloxacin [27]. Experts classified the bactericidal activity as moderate for levofloxacin and high for moxifloxacin. The sterilizing activity of fluoroquinolones was judged to be moderate [28–30]. Experts determined that fluoroquinolones have a low propensity to acquire resistance.

Rifabutin is an oral drug that kills mycobacteria by inhibiting the *Mtb* RNA polymerase [31]. Toxicity was classified as low (score 1.5) [32]. Experts stated that rifabutin does not prolong the QTc interval. The cost of 1 month treatment is $57.6 [18]. The EBA of rifabutin is low (fall in log_10_CFU 0. 0.07 [33]) and sterilizing activity is very high [34]. Experts classified the bactericidal activity as moderate. Rifabutin has a low propensity to acquire resistance.

Bedaquiline is an oral drug that inhibits the *Mtb* ATP synthesis [35]. Experts classified the toxicity for bedaquiline as low (score 1). The risk of QTc interval prolongation during bedaquiline treatment is moderate. Cost of one month bedaquiline treatment is $65 [18]. The EBA of bedaquiline is low with a fall in log_10_CFU of 0.04 [36] in the first two days of a loading dose of 400mg/day. Experts classified the bactericidal activity as moderate and sterilizing activity as very high. Bedaquiline has a moderate propensity to acquire resistance.

Clofazimine is an oral drug that inhibits protein synthesis [37]. Experts classified the toxicity of clofazimine as relatively high (score 2.25), it has no effect on the QTc interval [38]. The monthly cost of clofazimine treatment is $29.96 [18]. Clofazimine has a very low EBA as clofazimine treatment does not result in a drop in CFU counts in the first week of treatment [39]. Experts classified the bactericidal activity as moderate and sterilizing activity as low, and it has a low propensity to acquire resistance.

Linezolid is an oral drug that inhibits protein synthesis [40]. The risk of toxicity caused by linezolid is high (score 3) [41]. Expert opinion stated that linezolid has no effect on the QTc interval. The cost is $42.7 per month of linezolid treatment [18], it has a moderate EBA with a 0.17 drop in log_10_CFU in the first two days of treatment [27]. Experts classified the bactericidal and sterilizing activity of linezolid as high and the propensity to acquire resistance as low.

Ethambutol is an oral first line drug that inhibits the *Mtb* cell wall synthesis [42]. Toxicity is low (score 1), with a risk of SAE of 0.5% [25]. Expert opinion stated that ethambutol has no effect on the QTc interval. One month of ethambutol treatment cost $2.19 [18]. Ethambutol has a low EBA with a 0.245 drop in log_10_CFU in the first two days of treatment [43]. Experts classified the bactericidal activity as moderate, and it has almost no sterilizing activity [34] and a moderate propensity to acquire resistance.

Pyrazinamide is an oral drug that disrupts plasma membranes [44]. Toxicity of pyrazinamide is low (score 1) based on a 2.8% risk of SAE on pyrazinamide treatment [25]. Experts judged that pyrazinamide treatment has no effect on the QT interval. One month of treatment cost $2 [18]. Pyrazinamide has very low EBA [43, 45] and very high sterilizing activity [34]. Experts classified the bactericidal activity as moderate. Pyrazinamide has a high propensity to acquire resistance.

Isoniazid is an oral drug that inhibits *Mtb* mycolic acid synthesis [46]. Experts classified the toxicity as low (score 1) for isoniazid and high-dose isoniazid (score 1.25). Experts judged that isoniazid has no effect on QTc interval. One month of treatment cost $0.61 for isoniazid and $1.22 for high-dose isoniazid [18]. Isoniazid has a very high EBA with a drop in log_10_CFU of 0.50 [34] and moderate sterilizing activity [34]. Experts classified the EBA of high-dose isoniazid also as high and viewed the bactericidal activity of isoniazid and high-dose isoniazid as high. It has a low propensity to acquire resistance.

The thioamides, ethionamide and prothionamide, are oral drugs that inhibit *Mtb* cell wall synthesis [47]. Thioamides have a high toxicity (score 3) with a 8.2% SAE risk [25]. Experts judged that thioamides do not influence the QTc interval. The cost of one month of treatment is $11.8 for ethionamide and $18.95 for prothionamide [18]. Experts classified the EBA and bactericidal activity of thioamides as moderate, the sterilizing activity as low. Thioamides have a moderate propensity to acquire resistance.

Carbapenems, imipenem-cilastatin and meropenem, are administered via daily infusion together with oral clavulanate. They inhibit peptidoglycan synthesis [48]. Experts classified carbapenems as moderately toxic (score 1.75). Experts stated that carbapenems do not affect the QTc interval. Carbapenems are expensive drugs with a cost of $439.2 for one month imipenem and $338.55 for one-month meropenem [18]. The EBA is very low with small increase in log_10_CFU in the first 14 days of imipenem treatment [49]. Experts classified carbapenems as having a low bactericidal activity. In combination with clavulanate, carbapenems have a high sterilizing activity [50]. Carbapenems have a low propensity to acquire resistance.

The aminoglycosides (amikacin, kanamycin, and capreomycin) and streptomycin, drugs which need to be administered by daily intramuscular injection, inhibit protein synthesis [51]. Of the aminoglycosides only amikacin is currently recommended for treatment of RR-TB. Amikacin and streptomycin treatment is highly toxic (score 3) with 7.3% of patients reporting SAE for both injectables [25]. Experts judged that amikacin and streptomycin have no effect on the QTc interval. The EBA of the injectable drugs is low, with a loss of log_10_CFU of 0.05 for amikacin [27] and 0.04 for streptomycin [27]. The bactericidal activity and sterilizing activity of amikacin and streptomycin are also low [34, 52]. Both amikacin and streptomycin have a low propensity to acquire resistance.

Cycloserine and terizidone are oral drugs that inhibit protein synthesis [53]. These drugs have a high level of toxicity (score 2.75 for cycloserine and 3 for terizidone with a 4.5% and 9.1% SAE risk in patients treated with cycloserine and terizidone, respectively) [25, 54]. Experts stated that these drugs do not influence the QTc interval. The cost of one month treatment is $32.7 for cycloserine and $219.6 for terizidone [18]. Experts classified the EBA, bactericidal and sterilizing activity as low, moderate, and low for both drugs respectively. These drugs have a moderate propensity to acquire resistance.

Para-aminosalicylic acid (PAS) is an oral drug that inhibits DNA precursor synthesis [55]. PAS treatment is highly toxic (score 3) as 12.2% of patients treated with PAS reporting SAE [25]. There is no data to suggest that PAS influences QT interval. One month of PAS treatment costs $122 [18]. PAS has a moderate EBA with a drop in log_10_CFU of 0.259 in the first two days of treatment [45]. Experts classified the bactericidal activity of PAS as low and its sterilizing activity as moderate. PAS has a moderate propensity to acquire resistance.

The nitroimidazo-oxazoles, delamanid and pretomanid are oral drugs that inhibit mycolic acid synthesis [56]. At time of the development of the treatment recommender, only delamanid was registered for RR-TB treatment in South Africa. Experts classified toxicity of delamanid as low (score 1). QT prolongation is also low [57]. One month of delamanid treatment costs $308.63 [18]. Delamanid has a low EBA (drop in log_10_CFU of 0.066 [27]), a low bacterial and moderate sterilizing with dose-dependent killing rates. At the highest dose of delamanid, its sterilizing activity is superior to isoniazid and equal to rifampicin [58]. Delamanid has a moderate propensity to acquire resistance.

### Selection and quantification of regimen features

Experts agreed that an effective treatment regimen should consist of four effective drugs i.e., drugs to which no resistance has been detected. Fourteen features were defined to characterize the treatment regimens in the model. Features one to six are the sum of features of the four drugs included in the regimen: the sum of EBA, bactericidal activity, sterilizing activity, toxicity, propensity to acquire resistance and cost. Feature seven to nine assess whether the regimen adheres to the principles to construct a TB treatment regimen developed by Van Deun *et al*. [59] which state that a regimen should contain 1) at least one core drug that has a high bactericidal and sterilizing activity, 2) at least one highly bactericidal companion drug, one moderately bactericidal companion drug, and two moderately sterilizing companion drugs (a single companion drug can satisfy multiple requirements) and 3) a combination of a core and companion drugs. Features 10 and 11 quantify either the number of interactions between drugs in the regimen synergistic (pyrazinamide and bedaquiline, pyrazinamide and clofazimine, pyrazinamide and delamanid [60, 61]) or antagonistic (moxifloxacin and rifampicin, bedaquiline and rifampicin [62, 63]). Feature 12 checks whether all four drugs have a different mechanism of action. Given the current goal to preferentially administer all-oral regimens, feature 13 indicates whether the regimen is an all-oral regimen. Because experts rejected regimens containing two or more drugs with a moderate to high QTc prolongation effect during feedback, feature 14 was added to evaluate whether the sum (with low, moderate, and high equaling 1, 2, 3 respectively) of the QTc prolongation of the drugs is greater than 3.

Binary features are directly used as input for the treatment recommender model, while the continuous features undergo two transformations. Each continuous feature is therefore represented twice in the final treatment regimen feature set. The continuous features are normalized from 0 to 1, where the regimens with the highest score and lowest score obtain a value of 1 and 0 for that feature respectively. The second transformation divides the feature range into four parts (very high, high, low, very low) and classifies each continuous feature withing these classes. Negative features, such as cost, toxicity, antagonism, and propensity to acquire resistance are inverted such that higher values always equal patient benefit. Given that the total range for features varies when regimens are excluded (due to resistance), these transformations are performed after eliminating regimens containing drugs to which resistance was detected.

Table 2 shows the 14 features (only showing the continuous features before transformation) of the 10 highest ranked regimens by the CDSS for a patient infected with a *Mtb* strain that is resistant to rifampicin, isoniazid, ethambutol, ethionamide, prothionamide, rifabutin, and streptomycin. The regimen ranked number 1 would be recommended, the other nine regimens ranked in the top 10 for this resistance profile are increasingly suboptimal treatment regimens and the 100^th^ ranked regimen is an indication of a poor regimen for this resistance profile. The recommendation probability is the probability that the treatment recommender would classify this regimen as appropriate.

**Table 2:** The 10 highest ranked regimens (plus the 100th ranked regimen) for a *Mtb* strain.

	Rank	1	2	3	4	5	6	7	8	9	10	100
	Treatment recommender recommendation probability	86%	83%	78%	76%	72%	67%	66%	64%	62%	61%	24%
	Treatment regimen	LFX LZD BDQ PZA	LFX LZD BDQ CFZ	LFX BDQ CFZ PZA	LZD MFX BDQ PZA	MFX BDQ CFZ PZA	LZD MFX BDQ CFZ	LFX LZD CFZ PZA	LZD MFX CFZ PZA	LZD BDQ CFZ PZA	MFX BDQ SM PZA	LFX MFX TZD PZA
**1**	Sum of early bactericidal activity	11	11	9	11	9	11	10	10	7	10	13
**2**	Sum of bactericidal activity	12	12	11	13	12	13	13	14	12	11	13
**3**	Sum of sterilizing activity	16	14	14	16	14	14	13	13	15	14	12
**4**	Sum of toxicity	6.0	7.25	5.25	6.25	5.5	7.5	7.25	7.5	7.25	6.25	6.25
**5**	Sum of propensity to acquire resistance	9	11	9	9	9	11	10	10	9	9	9
**6**	Regimen cost ($)	114	142	101	119	106	147	79	84	140	95	234
**7**	Contains a core drug*	True	True	True	True	False	True	True	True	True	False	False
**8**	Contains companion drugs*	True	True	True	True	True	True	True	True	False	True	True
**9**	Contains core and companion drugs	True	True	False	True	False	True	True	True	False	False	False
**10**	Number of synergistic drugs	1	0	2	1	2	0	1	1	2	1	0
**11**	Number of antagonistic drugs	0	0	0	0	0	0	0	0	0	0	0
**12**	4 different mechanisms of action	True	False	True	True	True	False	False	False	False	True	False
**13**	All oral drugs	True	True	True	True	True	True	True	True	True	True	True
**14**	No more than 3 QTc prolongation	True	False	False	False	False	False	False	False	False	False	True

Table containing the regimen features for the first 10 and the 100^th^ ranked regimen for a *Mtb* strain resistant to rifampicin, isoniazid, ethambutol, ethionamide, prothionamide, rifabutin, and streptomycin.

* Core and companion drugs as defined by Van Deun *et al*. [59]. BDQ: bedaquiline, CFZ: clofazimine, LFX: levofloxacin, LZD: linezolid, MFX: moxifloxacin, PZA: pyrazinamide, SM: streptomycin, TZD: terizidone

### Acceptance of the RR-TB treatment recommender model in a clinical trial setting

**Fig 2:**
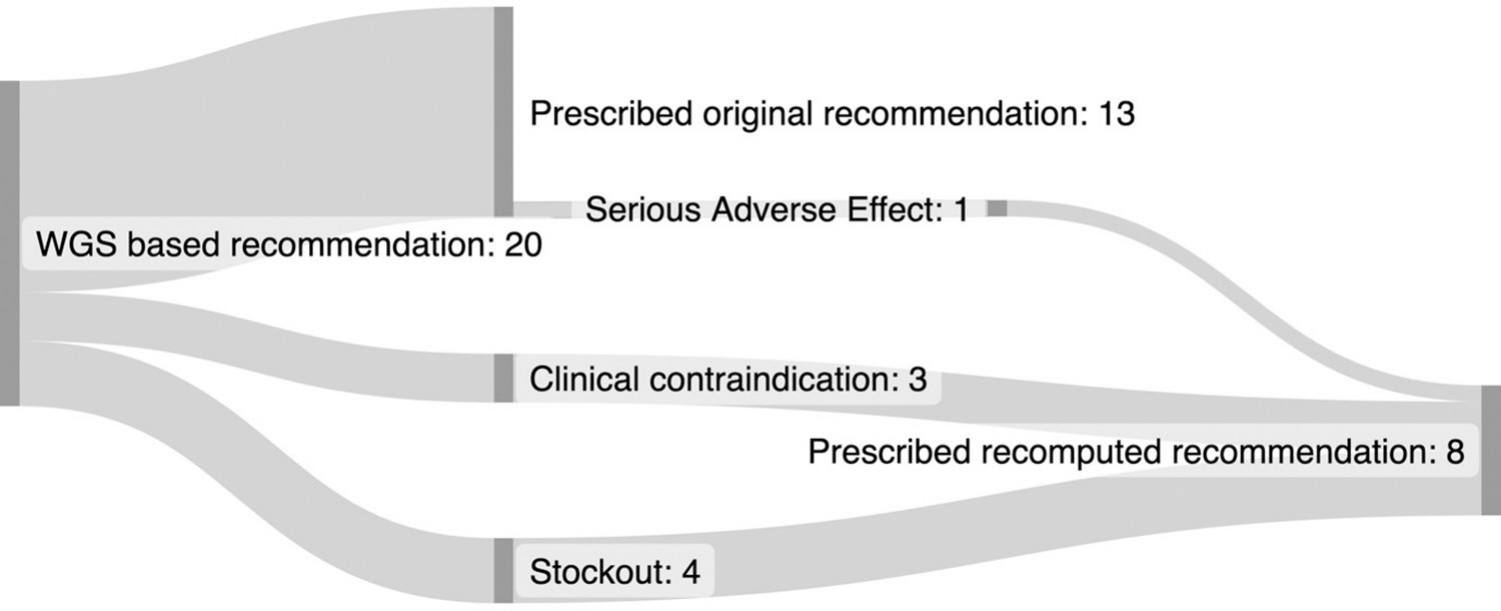
Treatment individualization flow for the first 20 patients in the SMARTT trial WGS arm.

Of the first 20 patients enrolled in the SMARTT trial for which the treatment recommender was used to recommend the individualized treatment regimen, all 20 (100%) patients received the regimen recommended by the treatment recommender CDSS when taking the *Mtb* resistance profile, clinical data of the patient and drug availability at the clinic into account. For 7 (35%) patients, the physician used the webapp to make changes to the initial recommendation had to be adjusted because of the presence of a clinical contra-indication for one of the drugs (n = 3) or because one of the drugs included in the recommended regimen was not available at the clinic (n = 4), information which was not available when the initial regimen was computed. For these seven patients the treatment recommender CDSS was rerun after the physician entered this information in the webapp, and these patients were prescribed the recomputed treatment regimen (Fig 2). For one patient, the regimen had to be recomputed at week 8 of individualized treatment due to a serious adverse effect to linezolid.

### Acceptance of treatment recommender CDSS in a routine care setting

All physicians agreed to prescribe the recommended regimen for 4 of the 15 profiles, and all physicians disagreed with 2 of the 15 profiles (Table 3). The reason for most rejections were deviations from national RR-TB treatment guidelines. The most common reasons were that the South African guidelines state that bedaquiline should not be combined with moxifloxacin and that group A drugs should always be combined with group B drugs. In addition, a regimen was rejected because the physician believed that resistance to isoniazid always occurs in case of ethionamide resistance, which is not the case.

**Table 3:** Acceptance of the CDSS recommendation for individualized RR-TB treatment in routine care setting.

resistance	treatment	Accept/Reject	Reason for rejection
RIF INH EMB ETH/PTH RBT SM	BDQ & LFX & LZD & PZA	5/0	
RIF INH EMB PZA ETH/PTH RBT SM	BDQ & CFZ & LFX & LZD	5/0	
RIF INH EMB PZA AMI/CAPREO/KANA RBT SM	BDQ & CFZ & DLM & LZD	5/0	
RIF INH EMB PZA ETH/PTH LFX/MFX RBT SM*	BDQ & CFZ & DLM & LZD	4/0	
RIF INH EMB PZA ETH/PTH RBT	BDQ & CFZ & LFX & LZD	4/1	No reason given
RIF INH EMB PZA RBT SM	BDQ & CFZ & LFX & LZD	4/1	No reason given
RIF INH EMB ETH/PTH RBT	BDQ & LFX & LZD & PZA	3/2	No WHO group B drugs
RIF INH EMB RBT SM	BDQ & LFX & LZD & PZA	3/2	No reason given
RIF ETH/PTH RBT	BDQ & INH & LFX & LZD	3/2	No WHO group B drugs and INH resistance expected due to ETH/PTH resistance
RIF INH EMB RBT	BDQ & LFX & LZD & PZA	2/3	No WHO group B drugs
RIF RBT	BDQ & EMB & LFX & PZA	1/4	Don’t combine MFX with BDQ
RIF INH ETH/PTH RBT	BDQ & CFZ & MFX & PZA	1/4	Don’t combine MFX with BDQ
RIF INH RBT	BDQ & CFZ & MFX & PZA	1/4	Don’t combine MFX with BDQ
RIF INH RBT SM	BDQ & CFZ & MFX & PZA	0/5	Don’t combine MFX with BDQ
RIF INH BDQ ETH/PTH RBT	CFZ & EMB & MFX & PZA	0/5	No WHO group B drugs

Hypothetical responses whether a physician would prescribe the regimen for 15 drug resistance profiles. An Asterix (*) indicates that a response was missing for one participant, AMI: amikacin, BDQ: bedaquiline, CAPREO: capreomycin, CFZ: clofazimine, DLM: delamanid, EMB: ethambutol, ETH: ethionamide, INH: isoniazid, KANA: kanamycin, LFX: levofloxacin, LZD: linezolid, MFX: moxifloxacin, PTH: prothionamide, PZA: pyrazinamide, RBT: rifabutin, RIF: rifampicin, SM: streptomycin.

### Acceptance of the treatment recommender CDSS system and its accompanying webapp

When using the modified technology acceptance model as a guide to assess the acceptance of the treatment recommender CDSS and its accompanying webapp (S2 Table), we found that the score was high (≥75%) for most domains except for the subjective norm (67%) and facilitators domain (58%) (Table 4). The facilitators domain scored low because physicians stated that their settings did not have the required infrastructure. Internet connection or access to email is not always available in more rural settings and they often use personal devices that do not have infrastructure support. The full questionnaire with answers is available in S2 Table.

**Table 4:** Acceptance of the treatment recommender CDSS by technology acceptance model domain.

Domain	Max score possible	Median Score (%)	Range (%)
Perceived usefulness	24	19 (79%)	16-24 (67%-100%)
Perceived ease of use	32	27 (84%)	21-28 (66%-88%)
Attitude	16	13 (81%)	12-15 (75%-94%)
Compatibility	12	9 (75%)	6-10 (50%-83%)
Subjective norm	24	16 (67%)	13-20 (54%-83%)
Facilitators*	12	7 (58%)	6-10 (50%-83%)
Habit	12	9 (75%)	8-10 (67%-83%)
Intention	12	9 (75%)	8-12 (67%-100%)

*One physician did not fill in question 12, the median score of the other physicians was used

## Discussion

When a patient is infected with an *Mtb* strain that is resistant to one or more of the drugs included in the standardized treatment regimen, has a contra-indication to one of the drugs, or experiences a side effect that requires one of the drugs to be stopped either temporarily or permanently, an individualized treatment should be initiated. Using a combination of machine learning and expert knowledge, we successfully developed a CDSS that automatically composes an individualized treatment that balances effectiveness, tolerability from a patient perspective, and feasibility from a health system perspective. The CDSS facilitates decentralized care, even for those RR-TB cases which are not eligible for a standard RR-TB regimen because of drug resistance, contra-indication or toxicity to one of the drugs in the standard regimen. The interactive online tool further maximizes the utility of the CDSS as it allows physicians to enter patient characteristics (toxicity or contra-indications for certain drugs) and relevant health system characteristics (registration of certain drugs in a specific country or temporary drug stock outs at a certain facility) in real time. The system also generates a pdf document that can be printed for paper record keeping. The report can be modified such that it is user-friendly, intuitive, and useful for health care workers with different levels of knowledge of genome sequencing or experience in treating DR-TB.

The acceptability of the CDSS was assessed in the context of the SMARTT clinical trial, where we found that, among the first 20 trial participants for whom the CDSS was used, all patients started a regimen recommended by the CDSS. In most (65%) trial patients the regimen recommended solely based on the WGS-derived drug resistance profile was started. In the other third of patients, the CDSS had to be run a second time because one of the drugs included in the regimen was out of stock, presence of a clinical contra-indication or development of toxicity. In such cases, the webapp can be used to re-run the model in real time, while the patient is with the physician. When assessing the acceptability of the treatment recommender outside of the trial setting, we found that physicians were hesitant to deviate from the guidelines. Implementation of the CDSS in routine care will thus need to be accompanied by updated guidelines to reflect new knowledge, such as the ability to safely combine moxifloxacin and bedaquiline [64], and to correct misconceptions, such as the belief that ethionamide resistance is always associated with isoniazid resistance. In a future version of the CDSS, notes addressing common misconceptions could be added to the recommended treatment regimen. Training physicians on new guidelines and new knowledge could further improve the acceptance of the models’ recommendations. From a digital technology perspective, acceptance of the treatment recommender CDSS and its app was high, with physicians indicating that they believe the treatment recommender is both easy to use and useful in clinical practice. The main concern for successful implementation in clinical practice was the infrastructure and training required.

Few treatment recommender systems have been developed for infectious diseases. The most notable example being HIV-ASSIST, a decision support tool for antiretroviral treatment recommendations [65]. The development process of HIV-ASSIST closely resembles that of the RR-TB treatment recommender CDSS. In HIV-ASSIST, all combinations of possible multi-drug regimens are ranked based on a “multi-attribute utility function” that considers utility weights (features) for the drugs and drug regimens based on national guidelines and clinical expertise. While there are great similarities, our treatment recommender CDSS is fundamentally different in that we used machine learning to iteratively learn the importance of features and relationships between the features while HIV-assist is based on a manually developed a multi-attribute utility function. A second example is the Medscape Drug Interaction Checker, however its functionality is limited only to drug-drug interactions [66].

The novel RR-TB treatment recommender CDSS has several important strengths. First, the features are comprehensive as they were identified by a diverse group of stakeholders, including patients and policy makers. Second, a machine learning model was used, which is ideal to identify the different level of importance of features of individual drugs and treatment regimens as well as the complex interactions between these features. Third, the implementation of the treatment recommender CDSS was assessed in a pragmatic clinical trial setting, which closely mimics real-life clinical practice in South Africa, a high RR-TB setting. Finally, the most important strength of the treatment recommender is the system is ‘future-proof’. Updates in knowledge on the genotype-phenotype associations can easily be used as the input of the drug resistance profile for the recommender system. Updates in knowledge of drug features (for example improved estimate of the incidence of toxicity) simply requires a modification to the drug features database without a need to re-develop the model. Similarly, adding a new drug requires little effort as only its drug features need to be quantified. The feedback harvested can then be reused for the development of the new model based on the updated features or inclusion of a new drug. Re-training the entire model is only needed when a new feature is added. Furthermore, when, in future, the treatment recommender would be used on a large scale, the treatment outcome of patients receiving CDSS-guided could be used to re-train and iteratively improve the model over time.

Several limitations to the treatment recommender should also be noted. First, there is no ‘truth’ for what constitutes the optimal individualized DR-TB treatment regimen, making it difficult to assess the performance of the treatment recommender. Second, there may be patient- or pathogen-related considerations, such as extent and type of disease, that are not yet fully captured in the model. The treatment recommender should thus be viewed as a CDSS tool and not a substitute for clinical judgement. Third, while the GRADE methodology is the recommended approach for summarizing evidence when making clinical practice recommendations, the knowledge base was developed using published and unpublished data available in 2019 complemented with expert opinion when data was scarce. Updating the feature values with new knowledge could improve the accuracy of the model. Fourth, the price of the different drugs can vary between regions, countries, and over time, which may need regional adaption of the feature values. Fifth, while we develop the CDSS to be as transparent as possible, the use of a machine learning model makes it difficult for users to discern which features drive the decisions. Sixth, the treatment recommender CDSS was developed between 2019 and 2021 to individualize and shorten treatment for all patient when the 9-month 7-drug oral regimen was the RR-TB regimen of choice. In the setting of BPaL(M), a treatment recommender CDSS would likely not recommend individualized treatment for all patients with RR-TB. Instead, the CDSS would be most useful to individualize treatment when resistance or contra-indications to bedaquiline, linezolid or pretomanid occur, or to strengthen the BPaL regimen to prevent amplification of resistance due to suboptimal adherence. A second version of the treatment recommender is being developed for use in the BPaLM era. Finally, our study is limited to the South African setting, therefore prospective studies should demonstrate the effectiveness of the treatment recommender CDSS in other high burden settings.

In conclusion, the treatment recommender and its accompanying online platform present a novel strategy for real-time user-friendly support for decentralized management of treating complex RR-TB patients. Global implementation of such a treatment recommender CDSS can help realize the goal of prescribing the most effective and least toxic treatment regimen for all patients suffering from DR-TB.

## Supporting information

S1 Table Key drug features. (XLSX)

S2 Table Modified technology acceptance model questionnaire. (XLSX)
